# Supplementary figures and images for: Recombinant Lactaptin Induces Immunogenic Cell Death and Creates an Antitumor Vaccination Effect in Vivo with Enhancement by an IDO Inhibitor
Source: Molecules. 2020 Jun 17;25(12):2804. doi: 10.3390/molecules25122804 (PMC7355630; doi:10.3390/molecules25122804)

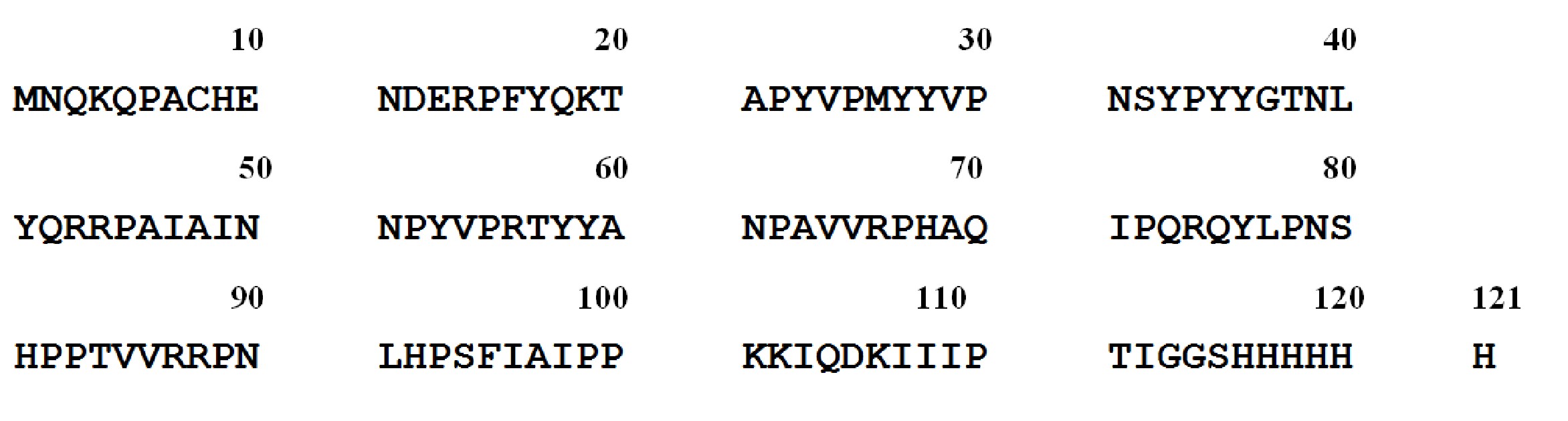

Supplement: Supplementary file 1 [file molecules-25-02804-s001.zip › molecules-830961-supplementary.JPG]
